# Psychological Capital Mediates the Association Between Perceived Organizational Support and Work Engagement Among Chinese Doctors

**DOI:** 10.3389/fpubh.2020.00149

**Published:** 2020-05-22

**Authors:** Shihan Yang, Hao Huang, Tian Qiu, Fangqiong Tian, Zhihui Gu, Xuege Gao, Hui Wu

**Affiliations:** ^1^Department of Social Medicine, School of Public Health, China Medical University, Shenyang, China; ^2^Department of Mathematics, University of California, San Diego, CA, United States

**Keywords:** Chinese doctors, work engagement, perceived organizational support, psychological capital, mediating effect

## Abstract

**Background:** As experts studying occupational health psychology know, low level of work engagement leads to higher turnover intentions. Some researchers have put a focus on the association between organizational support and work engagement. However, little has been done concerning the mediating effect of psychological capital (PsyCap) on the association between perceived organizational support (POS) and work engagement (vigor, dedication, absorption) among Chinese doctors.

**Methods:** A cross-sectional study has been carried out from November to December in 2017, in Liaoning Province, China. The questionnaire consists of Survey of Perceived Organizational Support, the Utrecht Work Engagement Scale, Psychological Capital Questionnaire, and demographic and working variables. The self-administered questionnaires were distributed to 1,009 doctors. Effective responses were collected from 836 participants (82.85%). Hierarchical multiple regression and the asymptotic and resampling strategies were used to examine the association between POS and work engagement mediated by PsyCap.

**Results:** After controlling the demographic and working variables, POS was positively related to vigor (β = 0.402, *P* < 0.01), dedication (β = 0.413, *P* < 0.01), and absorption (β = 0.373, *P* < 0.01). Psychological capital was positively associated with vigor (β = 0.442, *P* < 0.001), dedication (β = 0.413, *P* < 0.001), and absorption (β = 0.395, *P* < 0.001). Thus, PsyCap [a × b = 0.1895, bias-corrected and accelerated 95% confidence interval (BCa 95% CI) = 0.1524, 0.2290]; a × b = 0.1517, BCa 95% CI = 0.1180, 0.1875; a × b = 0.1693, BCa 95% CI = 0.1299, 0.2099] significantly mediated the association between POS and vigor, dedication, and absorption, respectively.

**Conclusion:** There was a low level of work engagement among Chinese doctors. Perceived organizational support could indirectly improve vigor, dedication, and absorption, partially through mediator PsyCap. Perceived organizational support intervention, education, and training in PsyCap should be carried out to cope with work engagement.

## Introduction

Work engagement is defined as an active, substantial, and job-relevant status of spirit (1). It consists of three subscales: vigor (i.e., always keep energetic work), dedication (i.e., actively and enthusiastically involved in the work), and absorption (i.e., totally focus on one's work) (2). Low work engagement among a large proportion of practitioners could generate a negative attitude toward their work (3). Around the world, low work engagement is a serious problem among all types of occupational groups, especially to the doctors (4–6). In China, shortage of doctors is a common phenomenon in the hospitals, which probably leads to overfatigue (7) and even low level of work engagement. The number of doctors was 2.83 million in 2017 in China, indicating the doctor–patient ratio is 2.44 doctors for 1,000 patients (8). This ratio is immensely lower than the ratio of 9.8 in high-income or the ratio of 4.45 in middle-income countries (9). More seriously, doctors in China complain about being in highly intensive work and in enormously stressful environment. Approximately 94% doctors work more than 8 h per day in China. One-quarter of doctors work more than 12 h per day without corresponding recompense. Most doctors must work on national holidays, who have not received contractually required income (10). In addition, doctors are often overworked. They have frequent night shifts, lots of daily consultations, and even hundreds of patients 1 day. This undoubtedly increases their occupational prevalence and undermines their work engagement compared with nurses and other occupations (4, 5, 11–13). Even worse, low work engagement may damage the job motivation and job enthusiasm of doctors and then exacerbate job burnout and turnover intentions (6, 14–16). Ultimately, a low level of work engagement has a negative impact on doctors' mental health and medical service quality (2). On the contrary, a high level of work engagement could promote doctors' working performance, contentment, and psychological health (17–19). Loerbroks et al. (2) research demonstrates that improving doctors' work engagement, particularly vigor and dedication, are related to better patient care. Therefore, work engagement of doctors in China needs further research.

A low level of work engagement dramatically impacts physical and psychological health, the quality of life, and the health service of the doctors. It is essential to find positive psychological resources to manage this adverse effect. Doctors are usually rewarded for providing excellent medical services. In the work, perceived organizational support (POS) could be maintaining an employee's feelings. Perceived organizational support relates to the degree that the organization treasures his/her contribution and concerns with his/her well-being (20). It appears through encountering benefits to employees taken by the organization. Perceived organizational support is also a vital element of the social interaction. It means employees trust that the organization will provide encouragements and value their achievements, and the organization trusts that employees will perform excellently at work (21). Empirical studies have shown that low POS leads to negative work attitude and performance. Low POS also negatively influences mental health and employee engagement (20, 22–24). Especially in hospitals, low POS results to bad consequences in doctors, such as frequent absenteeism, reduced productivity, and separation (20, 25, 26). Furthermore, it may have a negative impact on the quality of medical services. Perceived organizational support was found to be a positive factor relating with work engagement in professional managers and nurses before (11, 27, 28). Hence, improving POS may be a kind of means for promoting the doctors' professional performance (20).

Psychological science is becoming increasingly important at all aspects of society. Because POS has been explored at the organizational aspect, it is also an interesting topic to enhance the doctors' work engagement at the personal aspect. The psychological factors at the personal perspective can enrich interventions in the future. Based on the results of many previous studies, a vital notion derived from organizational psychological behavior is psychological capital (PsyCap). It is a positive resource and psychological force against “doctor pressure and resignation” (29–33). As an exploitable human resource, PsyCap can be suitably developed through training and intervention programs (34). As people become increasingly aware of the significance of positive psychological resources, organizations seek to promote physical and mental health of doctors via reinforcing psychological resources (35). In recent years, some studies have shown that PsyCap relates to POS and work engagement in nurse and staff (11, 29), while PsyCap is connected with professional identity among Chinese doctors (33). For instance, raising PsyCap has positively to do with POS and work engagement (11, 36). Additionally, a previous review presents that POS could influence work engagement through PsyCap but lacks empirical research (34). Nevertheless, to the best of our knowledge, PsyCap has not been entirely certified as a mediator between POS and working engagement, particularly among doctors in the Asian countries. Therefore, we want to add psychological resources to the model of doctors' work engagement and to explore the mediating role of PsyCap in “organization” and “individual.”

## Purpose of the Study

**Figure 1:**
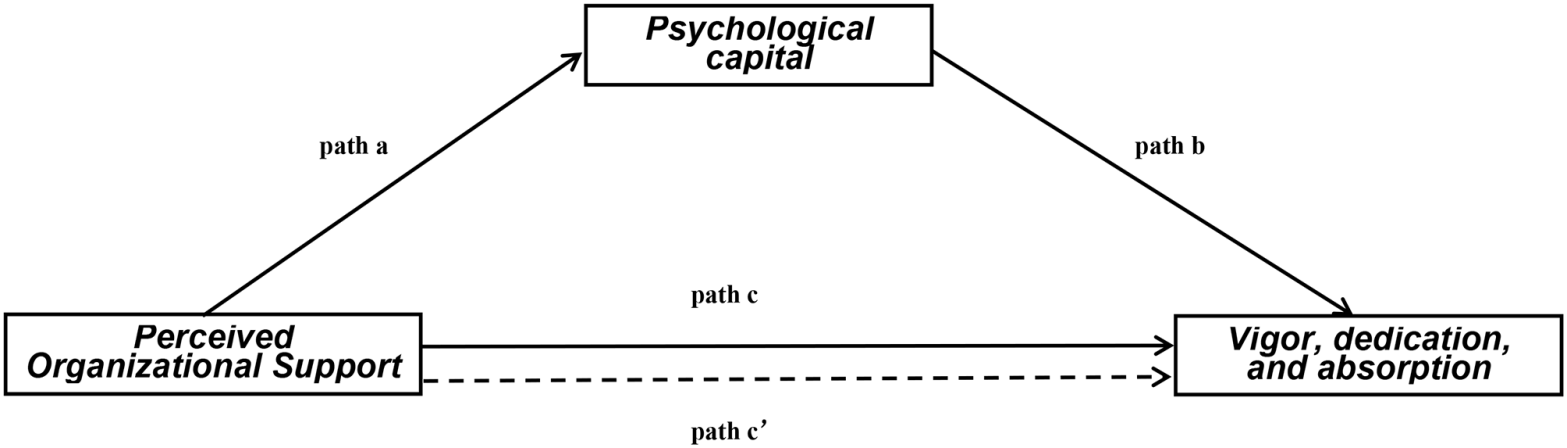
Theoretical model of the mediating role of PsyCap on the association between POS and work engagement. c: association of POS with vigor, dedication and absorption; a: association of POS with PsyCap; b: association between PsyCap and vigor, dedication, and absorption after controlling for the covariates; c′: association of POS with vigor, dedication, and absorption after adding PsyCap as a mediator; c = c′ + ab. POS, perceived organizational support; PsyCap, psychological capital.

Based on the research presented above, this study explored the potential mediating role of PsyCap on the association between organizational care and employee mentality. We try to explore the role of PsyCap on the relationships between POS and work engagement (vigor, dedication, and absorption) among Chinese doctors (Figure 1). Four hypotheses were proposed:

Hypothesis 1: Perceived organizational support is positively associated with work engagement (vigor, dedication, absorption) (path c).

Hypotheses 2 and 3: Psychological capital is positively related to POS (path a) and work engagement (vigor, dedication, absorption) (path b).

Hypothesis 4: The effects of POS on work engagement (vigor, dedication, absorption) are partially mediated by PsyCap (path c′).

## Materials and Methods

### Ethics Statement

All participants filled in the informed written consent voluntary and anonymous. The study was performed in accordance with the Declaration of Helsinki, and the protocol was approved by the Committee on Human Experimentation of China Medical University.

### Study Design and Sample

This cross-sectional study was conducted from November to December in 2017, in Liaoning Province, China. Liaoning province has 14 cities, 57 tertiary hospitals, and 98,985 doctors in 2017 (8). Tertiary hospitals need more than 500 beds in China (37). Based on geographic division, we selected doctors from five cities and randomly chose one tertiary hospital in each city. Then, we randomly selected ~200 doctors from each hospital. With acquiring informed consent written by participants, the self-administered questionnaires were distributed to 1,009 doctors. Eight hundred thirty-six doctors provided effective answers (effective response rate, 82.85%).

### Demographic Characteristics and Working Characteristics

Demographic characteristics included gender, age (years), marital status, and educational level. “Age (years)” was classified as ≤30, 31–40, and ≥41; “Marital status” was classified as single/widow/divorced/separated and marriage/cohabitation; “Educational level” was classified as junior college or lower, college, and postgraduate or higher. Working characteristics included professional title and monthly income (RMB, yuan); “Professional title” was classified as junior, intermediate, and senior; “Monthly income (RMB, yuan)” was classified as <4,000 yuan, 4,000 to 8,000 yuan, and >8,000 yuan.

### Measurement of Work Engagement

The Utrecht Work Engagement Scale (UWES) was used to assess work engagement. It has three subscales and 17 items, which are vigor (6 items), dedication (5 items), and absorption (6 items) (11). All items range from 0 (never) to 6 (always), and the total score of each dimension was valued by summing scores of each dimension (38). Higher scores indicate high-level work engagement. The Chinese version of UWES is practiced in many Chinese occupational groups. It has satisfactory reliability and validity (39, 40). In this study, Cronbach α coefficients for vigor, dedication, absorption, and the UWES scales were 0.848, 0.867, 0.835, and 0.943, respectively. The confirmatory factor analyses for the UWES were RMSEA (root mean square error of approximation) = 0.052, CFI (comparative fit index) = 0.979, GFI (goodness-of-fit index) = 0.962, AGFI (adjusted goodness-of-fit index) = 0.931, TLI (Tucker–Lewis index) = 0.966, and NFI (normed fit index) = 0.970.

### Measurement of POS

A 9-item scale of the Survey of Perceived Organizational Support (SPOS) was used to evaluate POS (20). All items were scored from 1 (strongly disagree) to 7 (strongly agree). Higher POS needs higher scores. The short-version SPOS is practiced in many Chinese occupational groups. It has satisfactory reliability and validity (23, 24). In this study, Cronbach α coefficient for the SPOS scale was 0.883. The confirmatory factor analysis for SPOS were RMSEA = 0.045, CFI = 0.991, GFI = 0.982, AGFI = 0.968, TLI = 0.987, and NFI = 0.985.

### Measurement of PsyCap

Psychological Capital Questionnaire (PCQ) was used to examine PsyCap, which has 24 items (41). All items were scored from 1 (strongly disagree) to 6 (strongly agree). Higher PsyCap needs higher scores. Numerous Chinese researches have used the Chinese version of the PCQ and have excellent reliability and validity (42–44). In this study, Cronbach α coefficient for PsyCap scale was 0.934. The confirmatory factor analyses for PCQ were RMSEA = 0.049, CFI = 0.962, GFI = 0.940, AGFI = 0.917, TLI = 0.951, and NFI = 0.945.

### Statistical Analyses

The demographic and working variables were described with mean ± SD. Differences of the mean value of work engagement in different groups were tested by *t* test or one-way analysis of variance. Pearson correlation analysis was used to assess correlations among continuous variables. The validity of the scales was tested by confirmatory factor analysis. In this study, multicollinearity was not an issue in the estimate because of variance inflation factor values <10. Hierarchical multiple regression was applied to explore the mediating effect of PsyCap in the association between POS and work engagement. All variables in univariate analysis (*P* < 0.05) were entered: Step 1, adding covariates demographic and working variables (gender, age, and professional title); Step 2, adding independent variable POS; Step 3, adding mediation PsyCap. As shown in Figure 1, in Steps 1 and 2 after adjusting for covariates, the purpose was to test whether POS has an effect on work engagement (the c path). In Step 3, the purpose was to explore the mediation of PsyCap. If the effect of POS on work engagement (c′ path coefficient) in Step 3 was smaller than the c path coefficient in Step 2, PsyCap was likely to be considered to have a partial mediating role (45, 46). Asymptotic and resampling strategies were used to examine PsyCap as potential mediator in the association between POS and work engagement (vigor, dedication, and absorption) based on 5,000 bootstrap samples. The bias-corrected and accelerated 95% confidence interval (BCa 95% CI) was estimated for mediation, and a BCa 95% CI excluding 0 indicated a significant mediating role (37). All the above analyses were conducted using IBM SPSS Statistics 21.0 (IBM, Asia Analytics Shanghai, China) and IBM AMOS 21.0 (IBM, Asia Analytics Shanghai, China) statistical software for Windows. Two-tailed *P* < 0.05 was viewed as statistically significant in this study.

## Results

### Demographic and Working Characteristics of Subjects of Work Engagement

Demographic and wok characteristics of participants and comparisons on vigor, dedication, and absorption are shown in Table 1. The score of absorption in women's group was significantly higher than that in men's group (*P* < 0.01). The scores of vigor, dedication, and absorption in the senior professional title group were significantly higher than those in junior and intermediate groups, respectively (*P* < 0.001).

**Table 1:** Demographic and working characteristics of subjects and comparisons on vigor, dedication, and absorption.

**Variables**	***n* (%)**	**Vigor Mean ± SD**	***F*/*t***	***P***	**Dedication Mean ± SD**	***F*/*t***	***P***	**Absorption Mean ± SD**	***F*/*t***	***P***
Gender			0.472	0.637		1.856	0.064		2.752	0.006
Men	285 (34.1)	23.64 ± 8.32			21.13 ± 7.12			23.74 ± 8.79		
Women	551 (65.9)	23.92 ± 7.84			22.06 ± 6.70			25.42 ± 7.50		
Age (years)			1.637	0.195		1.175	0.309		1.948	0.143
≤30	162 (19.4)	24.01 ± 7.44			21.72 ± 5.99			24.30 ± 7.48		
31–40	467 (55.9)	23.41 ± 8.09			21.48 ± 7.09			24.63 ± 7.92		
≥41	207 (24.8)	24.60 ± 8.19			22.35 ± 6.94			25.77 ± 8.52		
Marital status			0.507	0.613		0.021	0.983		0.224	0.823
Single/widow/divorced/separated	148 (17.7)	24.13 ± 8.16			21.75 ± 6.78			24.98 ± 7.58		
Marriage/cohabitation	688 (82.3)	23.76 ± 7.97			21.74 ± 6.88			24.82 ± 8.09		
Educational level			1.715	0.181		2.743	0.065		1.980	0.139
Junior college or lower	60 (7.2)	25.26 ± 8.59			23.03 ± 7.30			25.56 ± 9.19		
College	138 (16.5)	22.99 ± 7.34			20.70 ± 7.12			23.65 ± 8.11		
Postgraduate or higher	638 (76.3)	23.87 ± 7.99			21.84 ± 6.74			25.04 ± 7.84		
Professional title			9.755	0.000		9.120	0.000		7.688	0.000
Junior	300 (35.9)	24.27 ± 7.56^b^			22.21 ± 6.40^b^			24.68 ± 7.46^b^		
Intermediate	401 (48.0)	22.73 ± 8.12			20.80 ± 7.11			24.17 ± 8.30		
Senior	135 (16.1)	26.08 ± 8.10^a^			23.50 ± 6.68^a^			27.24 ± 7.83^a^		
Monthly income (RMB, yuan)			0.393	0.675		0.578	0.556		0.857	0.425
<4,000	80 (9.6)	24.17 ± 7.82			21.75 ± 6.21			24.94 ± 7.65		
4,000–8,000	286 (34.2)	23.49 ±± 8.02			21.39 ± 7.04			24.35 ± 7.68		
>8,000	470 (56.2)	23.97 ± 8.03			21.95 ± 6.83			25.13 ± 8.25		

a,b *Significantly higher compared with intermediate group, P < 0.001*.

*SD, standard deviation*.

### Correlations Among Study Variables

Correlations among study variables are shown in Table 2. The mean age of our sample was 36.55 (SD = 7.31) years, and the mean scores of vigor, dedication, and absorption were 23.82 (SD = 8.00), 21.74 (SD = 6.86), 24.85 (SD = 8.00), respectively. Age was positively correlated with PsyCap. Perceived organizational support was positively connected with PsyCap. Perceived organizational support and PsyCap were positively connected with vigor, dedication, and absorption among Chinese doctors.

**Table 2:** Correlations among study variables.

**Variable**	**Mean ± SD**	**1**	**2**	**3**	**4**	**5**	**6**
1. Age	36.55 ± 7.31	1					
2. POS	44.18 ± 7.96	0.000	1				
3. PsyCap	104.83 ± 13.5	0.803^*^	0.424^***^	1			
4. Vigor	23.82 ± 8.00	0.705^*^	0.403^***^	0.535^***^	1		
5. Dedication	21.74 ± 6.86	0.602	0.416^***^	0.514^***^	0.862^***^	1	
6. Absorption	24.85 ± 8.00	0.105^**^	0.376^***^	0.485^***^	0.846^***^	0.820^***^	1

* *P < 0.05*,

** *P < 0.01*,

*** *P < 0.001 (two-tailed)*.

*SD, standard deviation; POS, perceived organizational support; PsyCap, psychological capital*.

### Associations of POS and PsyCap With Work Engagement

In Table 3, the hierarchical regression analysis was performed to investigate the contribution and mediation associated with work engagement (vigor, dedication, and absorption). In Step 1, we found that gender and professional title were associated with work engagement. In Step 2, after controlling for gender, age, and professional title, POS was positively related to work engagement [vigor (β = 0.402; *P* < 0.001), dedication (β = 0.413; *P* < 0.001), and absorption (β = 0.373; *P* < 0.001)], explaining 16.1, 17.0, and 13.8% of the variance of vigor, dedication, and absorption, respectively. In Step 3, PsyCap was positively associated with work engagement [vigor (β = 0.442, *P* < 0.001), dedication (β = 0.413, *P* < 0.001), and absorption (β = 0.395, *P* < 0.001)], explaining 15.9, 13.8, and 12.7% of the variance of vigor, dedication, and absorption, respectively. Moreover, the positive effect of POS on work engagement (0.214, 0.237, 0.204) in Step 3 was smaller than that (0.402, 0.413, 0.373) in Step 2, indicating the probable mediation of PsyCap in the relationship between POS and work engagement (vigor, dedication, and absorption).

**Table 3:** Associations of POS and PsyCap with vigor, dedication, and absorption.

**Variables**	**Vigor**			**Dedication**			**Absorption**		
	**Block 1 (β)**	**Block 2 (*β)***	**Block 3 (*β)***	**Block 1 (*β)***	**Block 2 (*β)***	**Block 3 (*β)***	**Block 1 (*β)***	**Block 2 (*β)***	**Block 3 (*β)***
Gender	0.021	0.002	0.021	0.068	0.048	0.066^*^	0.108^**^	0.090^**^	0.108^***^
Age (years)	0.102^*^	0.091^*^	0.068	0.095	0.083	0.062	0.100^*^	0.089	0.068
Professional title	−0.035	−0.022	−0.038	−0.039	−0.025	−0.041	0.017	0.030	0.015
POS		0.402^***^	0.214^***^		0.413^***^	0.237^***^		0.373^***^	0.204^***^
PsyCap			0.442^***^			0.413^***^			0.395^***^
*F*	1.886	41.959^***^	80.525^***^	2.593	45.464^***^	77.344^***^	6.388^***^	39.862^***^	67.016^***^
Adjusted *R*^2^	0.003	0.164	0.323	0.006	0.176	0.314	0.019	0.157	0.283
Δ*R*^2^	0.007	0.161^***^	0.159^***^	0.009	0.170^***^	0.138^***^	0.023^***^	0.138^***^	0.127^***^

* *P < 0.05*;

** *P < 0.01*;

*** *P < 0.001 (two-tailed). Gender, men vs. women. Age was controlled in the model as a continuous variable*.

*POS, perceived organizational support; PsyCap, psychological capital*.

## Mediating Role of PsyCap

Based on the results of hierarchical linear regression analysis in Table 3, asymptotic and resampling strategies were used to examine the mediating role of PsyCap. In Table 4, POS was positively associated with PsyCap (a = 0.7231, P < 0.001). Thus, PsyCap (a × b = 0.1895, BCa 95% CI = 0.1524, 0.2290) significantly mediated the association between POS and vigor; PsyCap (a × b = 0.1517, BCa 95% CI = 0.1180, 0.1875) significantly mediated the association between POS and dedication; PsyCap (a × b = 0.1693, BCa 95% CI = 0.1299, 0.2099) significantly mediated the association between POS and absorption.

**Table 4:** Mediating role of PsyCap.

**Dependent variables**	**Mediators**	**a**	**b**	**a × b (BCa 95% CI)**
Vigor	PsyCap	0.7231^***^	0.2621^***^	0.1895 (0.1524, 0.2290)
Dedication	PsyCap	0.7231^***^	0.2097^***^	0.1517 (0.1180, 0.1875)
Absorption	PsyCap	0.7231^***^	0.2341^***^	0.1693 (0.1299, 0.2099)

*** *P < 0.001 (two-tailed). Gender, age and professional title were adjusted. a: the association of POS with PsyCap; b: the association of PsyCap with vigor, dedication, and absorption after controlling for the covariates; a × b: the product of a and b; BCa 95% CI: the bias-corrected and accelerated 95% confidence interval*.

*PsyCap, psychological capital*.

## Discussion

In this research, we probed the relationships of POS and PsyCap with work engagement (vigor, dedication, absorption). Likewise, we examined the partially mediating role of PsyCap in the association between POS and work engagement (vigor, dedication, absorption) among Chinese doctors.

The present research results have theoretical and practical significance for work engagement. Perceived organizational support was discovered to be positively connected with work engagement (vigor, dedication, absorption). It is consistent with previous studies (11, 24). One of the proper explanations is: POS not only improves work attitude, but also promotes many positive organizational behaviors (11). The doctors who have been recognized by the organizations generally hold an organized identity themselves, which increases emotional bond with the organization (47). Positive and satisfactory work experience of doctors can promote their work actively. It can also improve their emotional response and attitude toward treatment (48), such as high work engagement. In this study, POS was positively connected with work engagement (vigor, dedication, absorption), which support our hypothesis. Therefore, hospital administrators should take some targeted interventions immediately to improve the organizational support of doctors, for example, creating a better working environment for doctors (49).

In particular, previous studies viewed PsyCap as a positive resource for countering negative health outcomes, such as “workplace pressure, lassitude, and work–family conflict” (35, 42–44, 50, 51). Luthans et al. (32) reported that high-level PsyCap can strengthen self-confidence, so that employees make their efforts to succeed. Bonner et al. (31) thought PsyCap as an antecedent to work engagement. Psychological capital can maintain one's ambition to accomplish goals and boost the positive psychological ability to deal with difficult problems (52, 53). Other researchers are also concerned about the potential role of PsyCap among doctors (33, 54, 55). Qiu et al. (33) showed workplace violence will reduce doctors' PsyCap level, as well as result in low-level professional identity. Another study showed that workplace bullying can lead to emotional exhaustion. It is exacerbated when psychological distress is too high (56). Once you have a psychological breakdown, you are failing to resist workplace bullying, and your work engagement is worse. Psychological capital, as a positive psychological resource, may play a positive role in resisting workplace violence and bullying and improving work engagement. Therefore, it is particularly important to strengthen PsyCap in Chinese doctors. In our study, the result explains the positive relationship between PsyCap and vigor, dedication, absorption among Chinese doctors. So, we have reason to think that PsyCap is a positive resource for improving work engagement (vigor, dedication, absorption).

Our research also found that PsyCap partially mediated the relationship between POS and work engagement (vigor, dedication, absorption) among Chinese doctors. This proposed that POS might be good for PsyCap (11) and increase doctors' PsyCap, in order to improve doctors' work engagement (vigor, dedication, absorption). The conceivable explanation is that with high level of PsyCap doctors could resist heavier psychological burden. Therefore, they can improve their POS and balance work–family schedule (34). This suggests hospital administrators improve the access to organizational support to improve the doctors' POS. As work demands increase and work resources decrease, the medical environment is increasingly deteriorating. At this point, it is critical to provide an active work environment for employees (57). Establishing a supportive work environment can effectively improve the psychological health and work attitude of doctors, as well as improve organizational performance.

Our findings provide empirical support for positive psychology about work engagement in Chinese doctors. Above all, our results have implications for intervening low level of work engagement in Chinese doctors. It is essential to enhance work engagement by proposing targeted measures in Chinese doctors. First, the impact of organizational encouragement on personal PsyCap is important. Hospital administrators should create a supportive organizational climate to improve the POS and increase the professional happiness of doctors (58). Our findings can also help hospital managers to comprehend doctors' dedication (58). Second, doctors need positive psychological intervention to improve their work engagement, especially boost their PsyCap by valid methods (59). For instance, we can develop a PsyCap intervention (PCI) training model (60). Measures from both working environment and personal resources can improve the doctor's performance, efficiency, and physical and mental quality (5, 50, 59).

In addition, our previous researches focus on the positive psychological resources to improve the mental health of doctors. By strengthening PsyCap, it reduces the physical and mental fatigue of doctors. The emphasis of this study lies in organizational benefits; it further studies the positive psychological resources to promote the organizational behavioral health of doctors. On the basis of insufficient organizational support, strengthening PsyCap can accelerate the improvement of physical and mental health of doctors; promote healthy workplace behaviors, such as high levels of work engagement; and ultimately increase organizational benefits and improve medical care. This article integrates positive psychology and organizational behavior and uses positive psychological resources to mediate the relationship between organizational support and doctors' organizational behavior. It could be beneficial to the intervention for low-level work engagement and the promotion for workplace health in Chinese doctors.

Nevertheless, this article has several limitations that need to be explained. First, the cross-sectional study design is unable to prove temporal relationship, which requires a longitudinal study to improve. Second, the study was limited to hospitals and not others such as general practice. Later, we would like to extend the study areas in the research. Third, the sample appears to be skewed toward younger doctors; we will pay more attention to the work engagement of older doctors in future research. Fourth, self-reporting is commonly assumed to cause inaccuracy; it should be minimized by using some effective measures.

## Conclusion

The findings of this study demonstrate that work engagement (vigor, dedication, absorption) of doctors in China was comparatively low, particularly in the dedication subscale. First, this study links POS with work engagement in the doctor field in the context of Chinese hospitals. Second, on the organizational psychology perspective, our findings help to identify that PsyCap really fosters work engagement as well as mediates the relationship between POS and work engagement among Chinese doctors. Our findings also pave the way for interventions that aim to increase doctors' well-being and performance. Third, our results can be used to study further on how to make the interventions more targeted in Asian countries, such as China, by providing practice guidelines for hospital leaders. We consider boosting the doctors' work engagement by improving salary rewards, providing a safe and comfortable working environment, carrying out PCI activities, and increasing the PsyCap levels of doctors.

## Data Availability Statement

The datasets generated for this study are available on request to the corresponding author.

## Author Contributions

HW and SY designed the research and organized the investigation. SY and HH carried out data analysis. SY wrote the paper. TQ, FT, ZG, and XG provided assistance in interpreting and paper writing. All authors read and approved the final manuscript.

## Conflict of Interest

The authors declare that the research was conducted in the absence of any commercial or financial relationships that could be construed as a potential conflict of interest.
